# Knockdown of PTGS2 by CRISPR/CAS9 System Designates a New Potential Gene Target for Melanoma Treatment

**DOI:** 10.3389/fphar.2019.01456

**Published:** 2019-12-05

**Authors:** Giuseppe Ercolano, Paola De Cicco, Valentina Rubino, Giuseppe Terrazzano, Giuseppina Ruggiero, Roberta Carriero, Paolo Kunderfranco, Angela Ianaro

**Affiliations:** ^1^Department of Oncology UNIL CHUV and Ludwig Institute for Cancer Research Lausanne, University of Lausanne, Lausanne, Switzerland; ^2^Department of Pharmacy, University of Naples Federico II, Naples, Italy; ^3^Department of Translational Medical Sciences, University of Naples Federico II, Naples, Italy; ^4^Department of Science, University of Basilicata, Potenza, Italy; ^5^Bioinformatic Unit, Humanitas Clinical and Research Center, Rozzano, Italy

**Keywords:** CRISPR/CAS9, PTGS2, COX2, melanoma, cancer

## Abstract

CRISPR/Cas9 has become a powerful method to engineer genomes and to activate or to repress genes expression. As such, in cancer research CRISPR/Cas9 technology represents an efficient tool to dissect mechanisms of tumorigenesis and to discover novel targets for drug development. Here, we employed the CRISPR/Cas9 technology for studying the role of prostaglandin-endoperoxide synthase 2 (PTGS2) in melanoma development and progression. Melanoma is the most aggressive form of skin cancer with a median survival of less than 1 year. Although oncogene-targeted drugs and immune checkpoint inhibitors have demonstrated a significant success in improving overall survival in patients, related toxicity and emerging resistance are ongoing challenges. Gene therapy appears to be an appealing option to enhance the efficacy of currently available melanoma therapeutics leading to better patient prognosis. Several gene therapy targets have been identified and have proven to be effective against melanoma cells. Particularly, PTGS2 is frequently expressed in malignant melanomas and its expression significantly correlates with poor survival in patients. In this study we investigated on the effect of *ptgs2* knockdown in B16F10 murine melanoma cells. Our results show that reduced expression of *ptgs2* in melanoma cells: *i*) inhibits cell proliferation, migration, and invasiveness; *ii*) modulates immune response by impairing myeloid derived suppressor cell differentiation; *iii*) reduces tumor development and metastasis *in vivo*. Collectively, these findings indicate that *ptgs2* could represent an ideal gene to be targeted to improve success rates in the development of new and highly selective drugs for melanoma treatment.

## Introduction

Cutaneous melanoma is a complex genetic disease, resulting from the sequential accumulation ofmutations capable of driving transformation and, subsequently, progression of the disease (Shain et al., 2015). Oncogene-targeted drugs such as vemurafenib and dabrafenib for BRAFV600 mutated melanoma, and immune checkpoint inhibitors targeting immunomodulatory molecules such as ipilimumab, the anti-cytotoxic-T-lymphocyte associated protein 4 (CTLA-4), and nivolumab and pembrolizumab, the anti-programmed cell death 1 (PD-1) have completely revolutionized the treatment of advanced melanoma in recent years. All these agents have demonstrated a significant success in improving overall survival (OS) in patients with metastatic melanoma (Chapman et al., 2011; Hauschild et al., 2012; Topalian et al., 2014; Schadendorf et al., 2015; Schachter et al., 2017). However, related toxicity and emerging resistance are ongoing challenges (Winder and Viros, 2018). One advancement of overcoming resistance to immunotherapy is the use of combination therapies. Results from pre-clinical studies and clinical trials indicate that combinations of checkpoint inhibitors, either alone or combined with novel agents, may further contribute to the improvement of patient outcomes (Khair et al., 2019). An attractive option in cancer treatment is the combined of gene therapy and immunotherapy to achieve beneficial management in melanoma patients. Gene therapy appears to be an appealing option to enhance the efficacy of currently available melanoma therapeutics leading to better patient prognosis (Menezes et al., 2018). Several gene therapy targets have been identified and have proven to be effective against melanoma cells (Viola et al., 2013). However, more studies are necessary to effectively prioritize candidate therapeutic targets for melanoma treatments. Great interest is underway focusing on molecules whose mechanisms may operate within the tumor microenvironment (TME) and could be involved in immune escape and tumor evolution. For instance, cancer development and therapeutic response may relate to numerous pro-inflammatory mediators including cytokines, chemokines, growth factors, free radicals, prostaglandins (PGs), and proteolytic enzymes, which infiltrate the TME stimulating cancer cell proliferation and inhibiting their death (Todoric et al., 2016). Moreover, these factors might cause a failure of the novel anti-cancer therapies by inducing an immunosuppressive setting, which in turn leads to drug resistance (Martin et al., 2016). Particularly, increased expression of PG-endoperoxide synthase 2 (PTGS2) also known as cyclooxygenase-2 (COX-2), and its major metabolite PGE2 has been observed in many different types of cancer including melanoma (Becker et al., 2009; Wang and Dubois, 2010; Ko et al., 2017). PGE2 is the major prostanoid demonstrated to play a predominant role in promoting tumor formation, progression, and metastasis by acting directly on tumor cells and on tumor stromal cells (Nakanishi and Rosenberg, 2013). In addition, PGE_2_ is also responsible for inducing an immunosuppressive response within the TME by suppressing the accumulation and the activation of conventional dendritic cells while promoting the differentiation of myeloid derived suppressor cells (MDSCs) from bone marrow (BM) myeloid progenitor cells (Wang and DuBois, 2016). More recently, we have demonstrated that PTGS2 may be considered an useful diagnostic tool in defining melanoma malignancy. In fact, PTGS2 is frequently expressed in malignant melanomas and its expression significantly correlated with poor survival in patients (Denkert et al., 2001; Goulet et al., 2003; Lee et al., 2008; Panza et al., 2016). On the other hand, the expression of miR-143-3p, a transcriptional suppressor of PTGS2, inversely correlates with human melanoma progression confirming a key role for PTGS2 in malignant melanoma (Panza et al., 2018). More interestingly, it has been recently shown by our group that inhibition of PTGS2 enhances the anti-tumor activity of PD-1/PD-L1 based immunotherapy (Botti et al., 2017). Collectively, these findings indicate that PTGS2 could represent an ideal gene to be targeted to limit the resistance to immunotherapy as well as to enhance the effectiveness of checkpoint inhibitors in melanoma treatment.

CRISPR/Cas9 [clustered regularly interspaced short palindromic repeats (CRISPR) associated nuclease 9] is a novel innovative technology to quickly perform genetic manipulations which have been used to study gene function and their role in cellular fitness (Koike-Yusa et al., 2014; Meyers et al., 2017). More recently, the employment of the CRISPR/Cas9 technology in the study of the oncogenic events occurring during tumorigenesis, has revolutionized the research in cancer biology. Most importantly, CRISPR/Cas9 models can support the initial stages of drug discovery by contributing to a new and more effective portfolio of cancer targets (Guernet and Grumolato, 2017; Behan et al., 2019). The aim of our study was to investigate the consequences of *ptsg2* deletion in melanoma development and progression. To this purpose we used the CRISPR/Cas9 technology in B16F10 murine cells and demonstrated that selective *ptsg2* knockdown resulted in reduced proliferation, migration, and invasion ability of melanoma cells. Furthermore, the deletion of *ptsg2* impaired MDSCs differentiation *in vitro* and reduced tumor development and metastasis *in vivo*.

## Materials and Methods

### Cell Culture

The murine melanoma cells B16F10 were purchased and characterized from IRCCS AOU San Martino–IST (Genova, Italy), and were cultured in Dulbecco’s modified Eagle’s medium (DMEM) containing 10% fetal bovine serum (FBS), 2 mmol/L L-glutamine, 100 μmol/L non-essential amino acids, penicillin (100 U/ml), streptomycin (100 μg/ml), and 1 mmol/L sodium pyruvate (all from Sigma-Aldrich, Milan, Italy). Cells were grown at 37°C in a humidified incubator under 5% CO2.

### Knockdown of *ptgs2* With CRISPR-Cas9

A CRISPR knockdown kit against mouse *ptgs2* was purchased from Origene Technologies Inc. (Rockville, MD, USA). Transfections were performed as recommended by the manufacturer. Briefly, 3 × 10^5^ B16/F10 cells were seeded into six-well plates and maintained for 24 h. TransIT-X2 Transfection Reagent (Mirus Bio LLC, Madison, WI USA) was used at a final concentration of 2.4% together with a total of 2 μg plasmid (1 μg gRNA or empty-plasmid control with 1 μg donor) per well. TransIT-DNA complexes were made up in serum-free growth medium. Cells were maintained for 48 h before cells were returned to growth medium. Transfected cells were sub-cultured seven times before puromycin selection (1 μg/ml, Santa Cruz Biotechnology, CA). Selected cells (puromycin resistant) were screened for expression of *ptgs2* by quantitative real-time PCR (qPCR) and Western blot analysis.

### RNA Purification and qPCR

Total RNA was isolated from cells by use of the TRI-Reagent (Sigma-Aldrich, Milan, Italy), according to the manufacturer’s instructions, followed by spectrophotometric quantization as previously described (De Cicco et al., 2016). Final preparation of RNA was considered DNA- and protein-free if the ratio between readings at 260/280 nm was ≥1.7. Isolated mRNA was reverse-transcribed by use of iScript Reverse Transcription Supermix (Bio-Rad, Milan, Italy). The quantitative real-time PCR was carried out in CFX384 real-time PCR detection system (Bio-Rad, Milan, Italy) with specific primers (mPTGS2 5′-TACCCTCCTCACATCCCTGA-3′,5′-CCTGCTTGAGTATGTCGCAC-3′) using SYBR Green master mix kit (Bio-Rad, Milan, Italy). Samples were amplified simultaneously in triplicate in one-assay run with a non-template control blank for each primer pair to control for contamination or primer dimers formation, and the ct value for each experimental group was determined. The beta actin housekeeping gene was used as an internal control to normalize the ct values, using the 2^−ΔCt^ formula.

### Western Blotting Analysis

Whole cell protein was extracted with ice-cold lysis buffer supplemented with protease inhibitors, as detailed previously (Panza et al., 2015). Equal amounts of proteins were resolved on 10% Tris–glycine gels and transferred onto a nitrocellulose membrane. After blocking the nonspecific binding sites, the membrane was incubated with the primary antibody (PTGS2; cod: 160126; diluted 1:600, Cayman, MI, USA) at 4°C overnight. The membrane was then incubated with the appropriate peroxidase-conjugated secondary antibody and the immunoreactive bands were visualized using the enhanced chemiluminescence reagents. To verify equal protein loading, the membrane was stripped and reprobed with anti-GAPDH antibody.

### Growth Rate Analysis

Cell growth rate was determined by MTT (3-(4,3-(4,5-dimethylthiazol-2-yl)-2,5 diphenyltetrazolium bromide 5-dimethylthiazol-2-yl)-2, 5-diphenyltetrazolium bromide) assay. Briefly, cells were seeded onto 96- well plates (3 × 10^3^ cells/well) and incubated for 24, 48, and 72 h before adding 25 µl MTT (Sigma–Aldrich; 5 mg/ml in saline). Cells were then incubated for an additional 3 h at 37°C. After this time interval, cells were lysed and dark blue crystals were solubilized with a solution containing 50% N,N-dimethyl formamide and 20% sodium dodecyl sulfate with an adjusted pH of 4.5. The optical density of each well was measured with a microplate spectrophotometer (TitertekMultiskan MCC/340), equipped with a 620-nm filter.

### Wound Healing Assay

Cells were seeded in 12-well plates (2 × 10^5^ cells/well). Once the cells reached 90% confluency, a wound area was carefully created by scraping the cell monolayer with a sterile 200 μl pipette tip. After being washed three times with PBS, scratches including the flanking front lines of cells, were photographed (20-fold magnification). Subsequently, the cells were incubated at 37°C in 5% CO2. The width of the wound area was monitored with an inverted microscope at various time points. The wound area was measured using Image J software (LASV3.8, Germany).

### Transwell Migration Assay

A complementary Transwell migration assay was performed by employing a modified Boyden chamber (Corning, pore size 8 μm, NY, USA). Briefly, the chambers were placed into a 24-well plate and a total of 2 × 10^5^ cells were placed in the upper chamber of the Transwell in serum-free medium. Six hundred microliter of DMEM medium containing 10% FBS was added to the lower chambers to stimulate cell migration. After incubation for 24 h at 37 °C, the Transwell insert were removed from the plate and non-migrant cells on the upper side of the filter were detached with the use of a cotton swab. Filters were fixed with 70% ethanol and stained with 0.2% crystal violet. Cells on the undersides of the filters were observed under a microscope at a magnification of 10x. For quantification, photographs were captured from five random fields across wells, and the number of migrated cells was counted by using Image-J software.

### Invasion Assay

A Transwell insert with 8 μm pore size (Millipore, USA) was coated on the upper side with Matrigel (Becton Dickinson Labware, USA) for the invasion assay. Briefly, the chambers were placed into a 24-well plate and cells (2.5 × 10^5^/ml) were plated in the upper chamber in serum-free DMEM. After the incubation period (16 h), the filter was removed, and non-migrant cells on the upper side of the filter were detached with the use of a cotton swab. Filters were fixed with 4% formaldehyde for 15 min, and cells located in the lower filter were stained with 0.1% crystal violet for 20 min and then washed with PBS. The filters were examined microscopically, and cellular invasion was determined by counting the number of stained cells on each filter in at least four to five randomly selected fields.

### Clonogenic Assay

Cells (1 × 10^3^ cells/well) were seeded in six-well plates and were maintained in culture for 14 days. The medium was changed every 2 days. Then, cells were washed twice with PBS, fixed by 4% paraformaldehyde, and stained with 0.5% crystal violet and colonies containing more than 50 cells (established by microscopy) were counted manually. Images of the colonies were obtained using a digital camera.

### ELISA

CXCL1/KC plasma concentrations were evaluated using ELISA kits according to the manufacturer’s instruction (DuoSet ELISA, R&D systems, Minneapolis, MN, USA).

### Prostaglandin E2 Assay

PGE2 concentrations was evaluated in cell culture supernatants obtained from B16/CTRL, B16/PTGS2Δ, and B16/SCR. Prostaglandin E2 EIA kit (Cayman Chemicals, Ann Arbor, MI)) was used according to the manufacturer’s instruction.

### Preparation of Melanoma Cells Conditioned Medium

Wild-type B16/F10 (B16/CTRL) or *ptgs2* knocked-down B16F10 (B16/PTGS2Δ) or empty-plasmid control B16F10 (B16/SCR) melanoma cells (80% confluence) were growth with DMEM 10% FBS in a 100-mm diameter dish. After 24 h, the medium was changed. The medium was collected after 48 h, centrifuged at 1,800 rpm for 10 min, and filtered through a 0.22-μm syringe filter.

### *Ex Vivo* Generation of MDSCs

Bone marrow cells were obtained from femurs and tibias of C57BL/6 mice, and the red blood cells were lysed. One million cells were seeded into 24-well plates in RPMI 1640 medium supplemented with 10% FBS, 10 ng/ml GM-CSF, 10 ng/ml IL-4, and 2-ME alone or in the presence of 30% v/v of B16/CTRL or B16/PTGS2 or B16/SCR conditioned medium (CM) (Youn et al., 2008). Celecoxib (20µM) was added to some wells containing 30% v/v of B16/CTRL CM at the beginning of the culture. The cultures were maintained at 37°C in 5% CO_2_-humidified atmosphere. On day 3 of culture, floating cells were gently removed, and fresh medium with cytokines and the respective CM was replaced. Cells were collected on day 5 and analyzed by flow cytometry. For MDSC staining, cells were incubated with allophycocyanin-anti-CD45, FITC-anti-Ly6C, PerCP-Cy5.5-anti-CD11b, and PE-anti-Ly6G antibodies for 30’ at 4°C. Flow cytometry and data analysis were performed by using a two-laser equipped FACSCalibur apparatus and the CellQuest analysis software (Becton Dickinson, Mountain View, CA).

### Mice

The experimental procedures, according to Italian (DL 26/2014) and European (n. 63/2010/UE) regulations on the protection of animals used for experimental and other scientific purposes, were approved by the Italian Ministry. All studies involving animals are reported in accordance with the ARRIVE guidelines for reporting experiments involving animals (Kilkenny et al., 2010). Mice were observed daily and humanely euthanized by CO_2_ inhalation if a solitary subcutaneous tumor exceeded 1.5 cm in diameter or mice showed signs referable to metastatic cancer. All efforts were made to minimize suffering. Female C57BL/6 mice (6 weeks old; 18−20 g) were purchased from Charles River Laboratories, Inc. Mice were housed at the Animal Research Facility of the Department of Pharmacy of the University of Naples Federico II.

### *In Vivo* Melanoma Model

In the cutaneous melanoma tumor model, mice were subcutaneously injected in the right flank with B16/F10, B16/PTGS2Δ, and B16/SCR cells (1 × 10^5^/0.1 ml) (n = 8 each group). Tumor growth was monitored every 3 days. Solid tumors formed at the site of injections were measured using a digital caliper, and tumor volume was calculated using the following equation: tumor volume = Π/6 (D1 × D2 × D3) where D1 = length; D2 = width; D3 = height and expressed as cm^3^. Tumor weight was evaluated post sacrification.

For tumor metastasis experiments, mice (n = 8 each group) were injected with B16/F10, B16/PTGS2Δ, and B16/SCR cells (5 × 10^5^/0.1 ml) *via* the tail vein. After 14 days, the mice were sacrificed and the lungs were removed and washed with PBS. The lung tumors were counted and lung images were captured.

### Preparation of a Single Cell Suspension From Tumor

Tumor-bearing mice were sacrificed 14 days after tumor implant and tumors were isolated. Single-cell suspensions from tumor tissue were prepared using the GentleMACS protocol (Miltenyi Biotec). Briefly, tumors were isolated and minced into small pieces followed by a mechanical dissociation step using the GentleMACS dissociator. Samples were then incubated for 40 min at 37°C with the following enzymes: collagenase I (10,000 U/ml) and dispase II (32 mg/ml). After a last mechanical disruption step, the digested tumors were harvested and filtered (over a 70 μM nylon filter, BD Falcon) and red blood cells were lysed by adding Tris-buffered ammonium chloride. Cells were then analyzed using flow cytometry.

### Flow Cytometry

Aliquots of 5 x 10^5^ cells were washed in FACS buffer (PBS, 0.1% BSA) and stained using the following panel of monoclonal antibodies (mAbs) to murine cell surface molecules (all from BD Biosciences): PerCP-Cy5.5-conjugated anti-CD11b, PE-conjugated anti-Ly6G, FITC-conjugated anti-Ly6C, allophycocyanin-conjugated anti-CD45. Cells were washed in FACS buffer and data were collected using a two-laser equipped FACSCalibur apparatus and the CellQuest analysis software (Becton Dickinson, Mountain View, CA).

### Public Gene Expression Analysis

Two gene expression datasets were collected and analyzed for PTGS2 expressions in human samples. The first public gene expression study came from a public microarray of normal skin and primary melanomas. Normalized gene expression, fold change, and *P* values were retrieved from Oncomine database (Rhodes et al., 2004). For the second gene expression dataset, RNA-sequencing data of formalin fixed primary human melanoma tissue sections were retrieved from GEO (GSE126076), as un-normalized data. Each single tumor was microdissected into samples containing tumor center, tumor border, or tissue adjacent to the tumor (Shurin et al., 2019). Differential expression analysis was performed using the edgeR package (Robinson et al., 2010), applying the TMM normalization method and the glmQLFTest function.

### Statistical Analysis

Data were expressed as the mean ± standard error of mean. Difference between groups was determined by one-way ANOVA. *P* < 0.05 was considered statistically significant.

## Results

### *PTGS2* Expression in Human Melanoma

**Figure 1:**
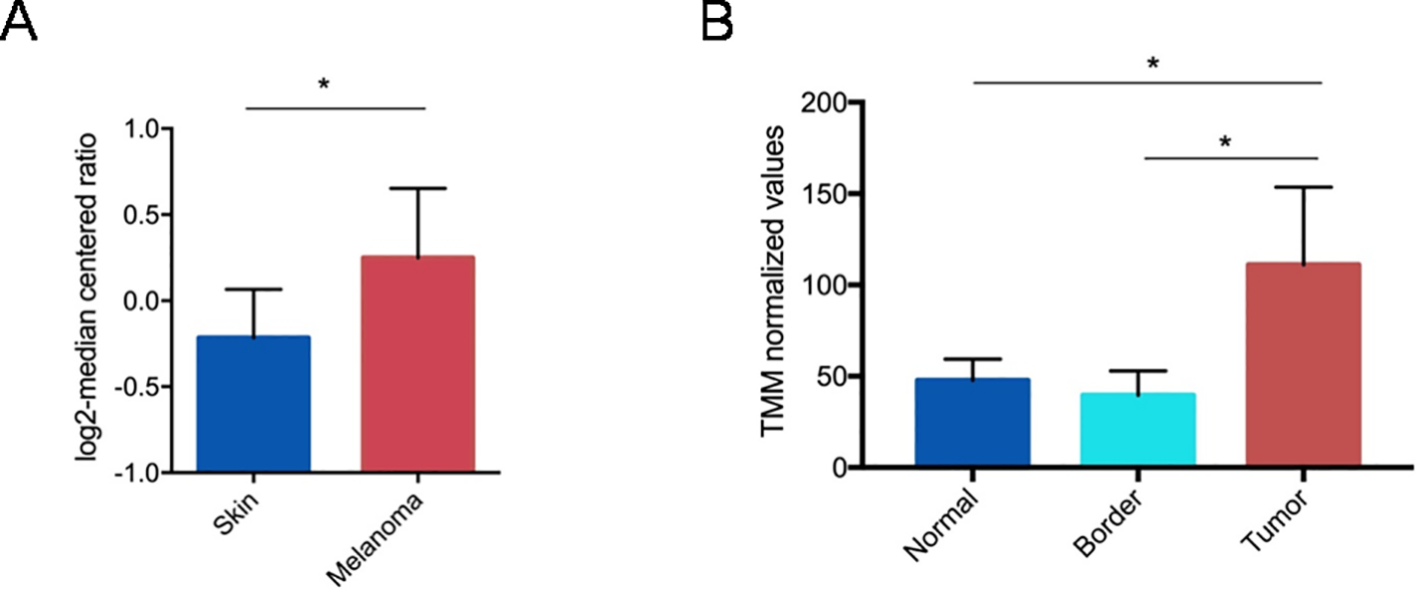
Gene expression analysis of *PTGS2* gene (R80217 probe) from Oncomine. Straight bars represent the median. The data are presented as the mean ± SEM. *PTGS2* resulted up-regulated in primary tumors respect to normal skin (fold change = 1.3; *P* = 0.046). (*P < 0.05) **(A)**. Gene expression analysis of *PTGS2* gene retrieved from GSE126076 dataset. Straight bars represent the median. The data are presented as the mean ± SEM. *PTGS2* resulted up-regulated in tumors respect to tumor borders (logFC = 1.43;FDR = 0.014) and normal tissues (logFC = 1.19; FDR = 0.012). *FDR < 0.05) **(B)**.

In order to investigate the correlation between *PTGS2* expression and human melanoma development we performed a bioinformatics analysis of available gene expression datasets. From Oncomine database, we found that *PTGS2* gene appeared to be up-regulated in primary tumors respect to normal skin (**Figure 1A**). To confirm this finding, PTGS2 expressions data in normal tissue, tumor mass, and tissue adjacent to the tumor were obtained from the dataset of Shurin et al. Similarly, we found that *PTGS2* resulted significantly up-regulated in tumor tissues compared to normal tissues and tumor border (**Figure 1B**). These evidence indicate that *PTGS2* has great potential to use as a biomarker and target for melanoma therapy.

### Generating *ptgs2* Knocked-Down Melanoma Cell Line Using the CRISPR/Cas9 System

**Figure 2:**
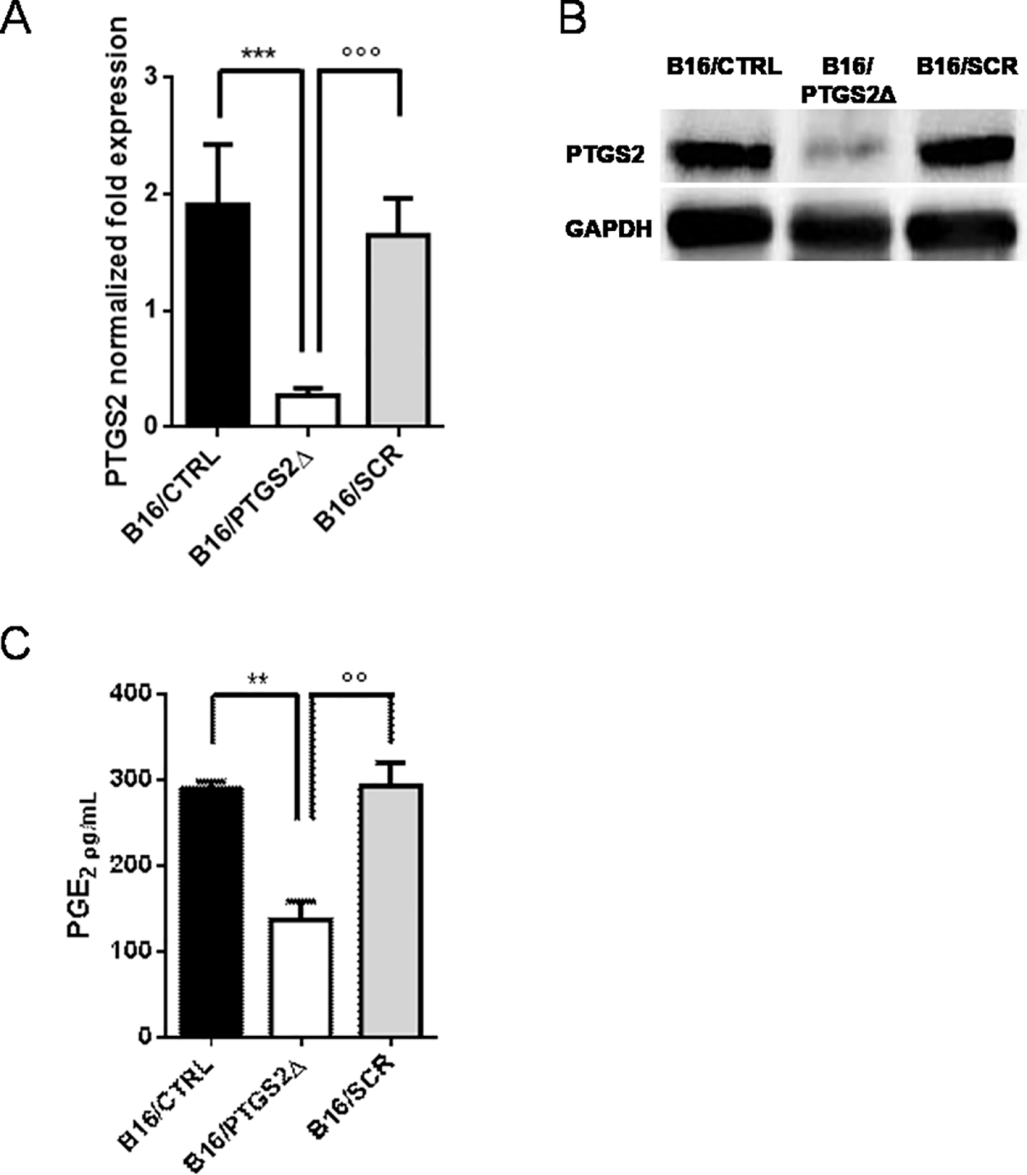
Knockdown of PTGS2 in B16/F10 melanoma cells. B16/F10 cells were transfected with PTGS2 CRISPR/Cas9 (B16/PTGS2Δ), or with empty-plasmid control (B16/SCR) or not transfected (B16/CTRL). Quantitative real-time PCR **(A)** and western blot analysis **(B)** showed significant PTGS2 knockdown in B16/F10 cells. PGE2 levels were measured in cell culture supernatants by EIA. PGE2 production by B16/PTGS2Δ was significantly reduced **(C)**. Data are shown as mean ± SEM of three independent experiments (**P < 0.01; ***P < 0.001 vs. B16/CTRL and °°*P* < 0.01; °°°*P* < 0.001vs. B16/SCR).

Next, we developed a cell line with a permanent knockdown of *ptgs2* (B16/PTGS2Δ) by using CRISPR/Cas9 technology, in order to study the effect of *ptgs2* deletion in melanoma development and progression. B16F10 murine melanoma cells were co-transfected with guide-RNAs targeting the *ptgs2* gene or scramble negative control (SCR) together with a Cas9 and a donor construct containing the puromycin resistance gene. We chose to delete one allele to assure a knockdown rather than a complete knockout, which might have been noxious. The knockdown of *ptgs2* was verified using qPCR (**Figure 2A**) and confirmed at the protein level by western blotting analysis (**Figure 2B**). The result showed that the expression of both PTGS2 mRNA and protein was significantly decreased in B16/PTGS2Δ cells compared to the corresponding vector control cells (B16/SCR) and to wild-type B16F10 cells (B16/CTRL). To further validate the efficiency of PTGS2 gene knockdown in B16F10 cells, we also measured the levels of PGE2 in cell supernatants. PGE2 is a potent inflammatory mediator that is generated by PTGS2 conversion of arachidonic acid. PGE2 is considered the cardinal mediator of PTGS2‐induced tumorigenesis. Indeed, it supports tumor growth by promoting angiogenesis, stimulating tumor-cell proliferation, protecting tumor cells from apoptosis, and inducing metastasis formation by upregulation of matrix metalloproteinases (MMPs) (Sinha et al., 2007). Production of PGE_2_ by *ptgs2* CRISPR-targeted clones was also greatly decreased (**Figure 2C**).

### *Ptgs2* Knockdown Reduces Cell Proliferation and Colony Formation

**Figure 3:**
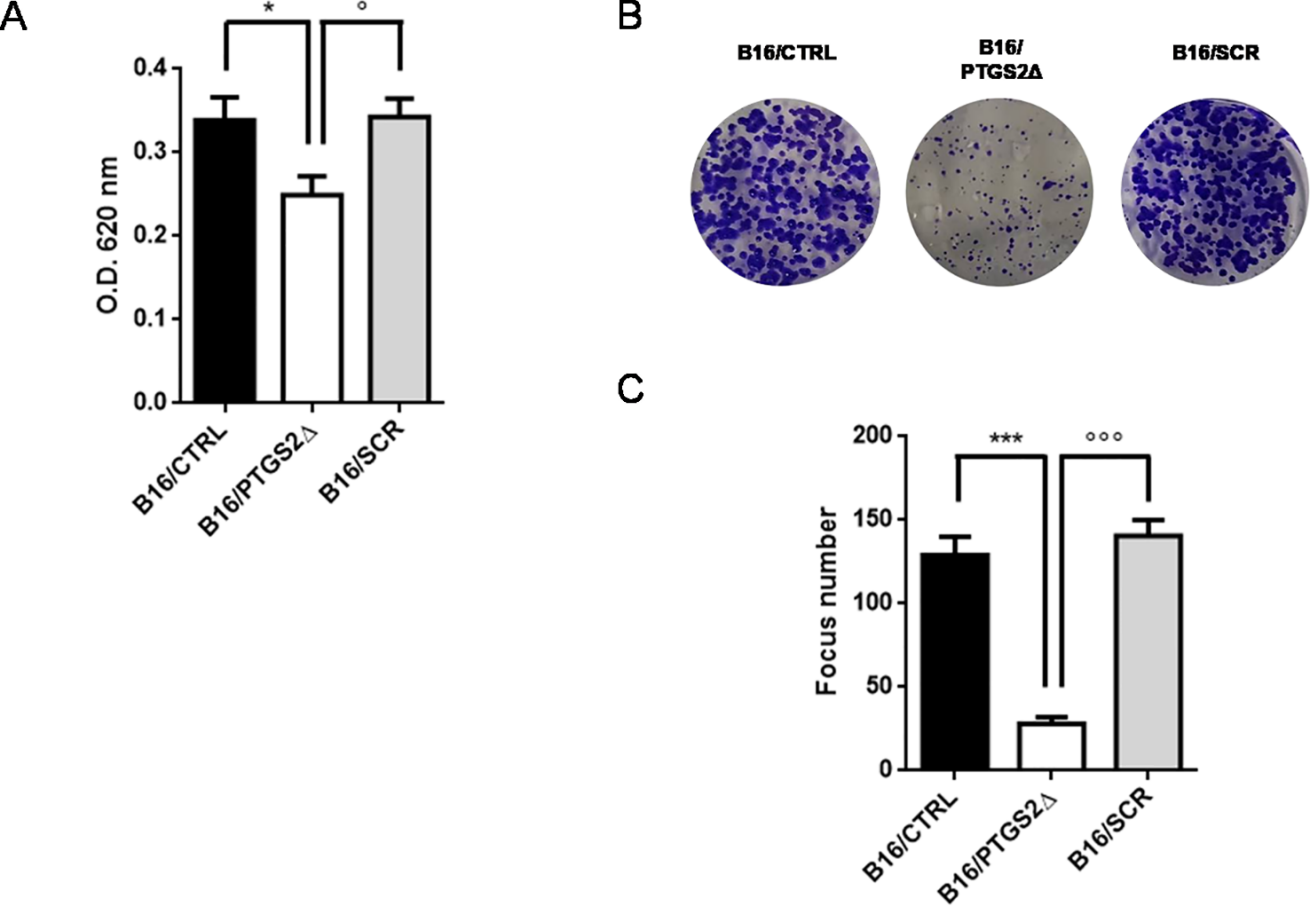
Knockdown of PTGS2 reduces cell proliferation and clonogenicity. Cell proliferation was determined by MTT assay and is expressed as OD values. B16/PTGS2Δ cells showed significantly slower proliferative rate than B16/CTRL or B16/SCR **(A)**. Clonogenic assay was performed on B16/F10 cells for 2 weeks. Representative images **(B)** and relative quantification of the colonies **(C)**. The number of colonies were fewer in B16/PTGS2Δ. Data are shown as mean ± SEM of three independent experiments (*P < 0.05 vs. B16/CTRL; °*P* < 0.05 vs. B16/SCR; ***P < 0.001 vs. B16/CTRL; °°°*P* < 0.001 vs. B16/SCR).

To address the role of PTGS2 in melanoma development, the potential effects of *ptgs2* knockdown on cell proliferation and colony formation were explored in B16F10 melanoma cells. The results obtained from the MTT assay demonstrated that B16/PTGS2Δ cells presented significantly slower proliferative rate than B16/CTRL or B16/SCR (**Figure 3A**). To further determine the effects of PTGS2 on cell proliferation, we performed a colony formation assay. Thus, B16/CTRL, B16/PTGS2Δ, and B16/SCR cells were seeded into six-well plates and incubated for 14 days to allow focus formation. As shown in **Figures 3B**, **C**, the focus diameter was smaller and the number of colonies were fewer in B16/PTGS2Δ group compared with B16/CTRL or B16/SCR.

### Ptgs2 Knockdown Reduces Cell Invasion and Motility

**Figure 4:**
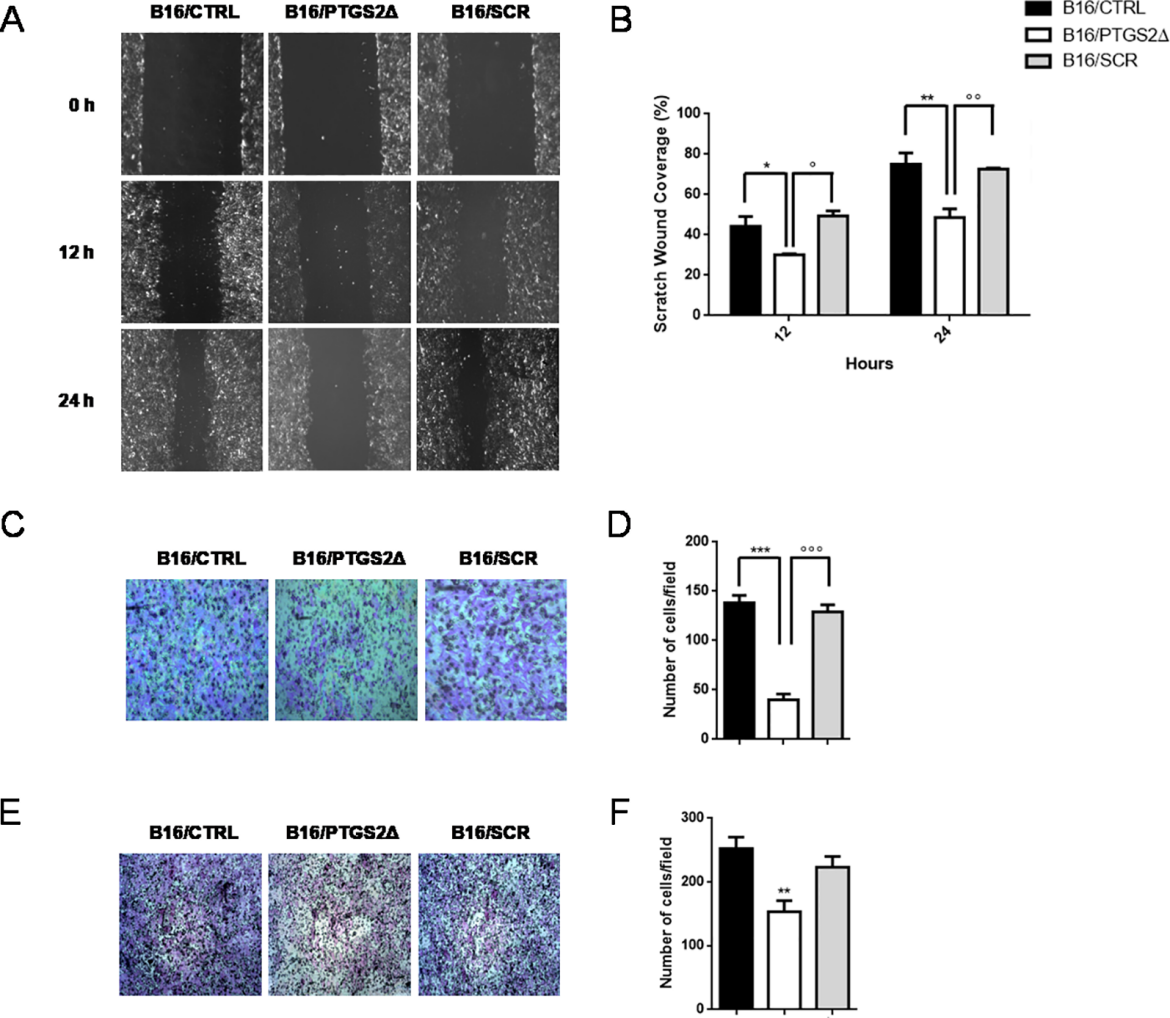
Knockdown of PTGS2 inhibits cell migration and invasion in B16/F10 melanoma cells. The migration potential of B16/PTGS2Δ was evaluated using the wound healing assay. Confluent B16/CTRL, B16/PTGS2Δ, and B16/SCR cells were wounded and allowed to migrate for 12 and 24 h. Cells at 0, 12, and 24 h were imaged **(A)** around wounds under phase contrast microscope (10×). Quantification of the healed wound area **(B)** demonstrated that B16/PTGS2Δ cells wound closure was significantly wider compared to B16/CTRL (*P < 0.05; **P < 0.01) and B16/SCR (°*P* < 0.05; °°*P* < 0.01). The motility of B16/PTGS2Δ was evaluated using a Transwell migration assay. Cells were seeded in the upper chamber and allowed to migrate for 24 h; then, the migrated cells were fixed and stained with crystal violet **(C)**, and the number of migrated cells was counted **(D)**. The average number of transmigrated B16/PTGS2Δ cells was lesser than B16/CTRL (***P < 0.001) and B16/SCR °°°*P* < 0.001). Cell invasion was determined using Boyden chambers coated with Matrigel. Representative images **(E)** and relative quantification of invaded cells **(F)**. The average number of invasive B16/PTGS2Δ cells was significantly reduced compared to B16/CTRL (**P < 0.01). All data are shown as mean ± SEM of three independent experiments.

Local cancer cell invasion and migration represent the crucial steps in the process of metastasis formation that dramatically worsen prognosis and patient’s survival (van Zijl et al., 2011). Thus, we then investigated whether the knockdown of *ptgs2* affected the motility and invasive properties of B16F10 cells. We first examined the effect on the migration of B16F10 cells in a scratch wound healing assay. Results showed that B16/PTGS2Δ displayed lower levels of migration than B16/CTRL and B16/SCR cells (**Figures 4A, B**), as the wound area 24 h after wound generation resulted almost 35% wider. Similar results were found in the Transwell assays, which showed that B16/PTGS2Δ cell line exhibited a reduced migratory capacity compared to B16/CTRL and B16/SCR cells (**Figures 4C, D**).

To evaluate cell invasion capacity, membranes were coated with Matrigel, mimicking the extracellular matrix. As show in **Figures 4E, F**, -B16/PTGS2Δ cells exhibited lower rate of invasion compared to B16/CTRL and B16/SCR cells.

### *Ptgs2* Knockdown Impairs MDSCs Differentiation *In Vitro*

**Figure 5:**
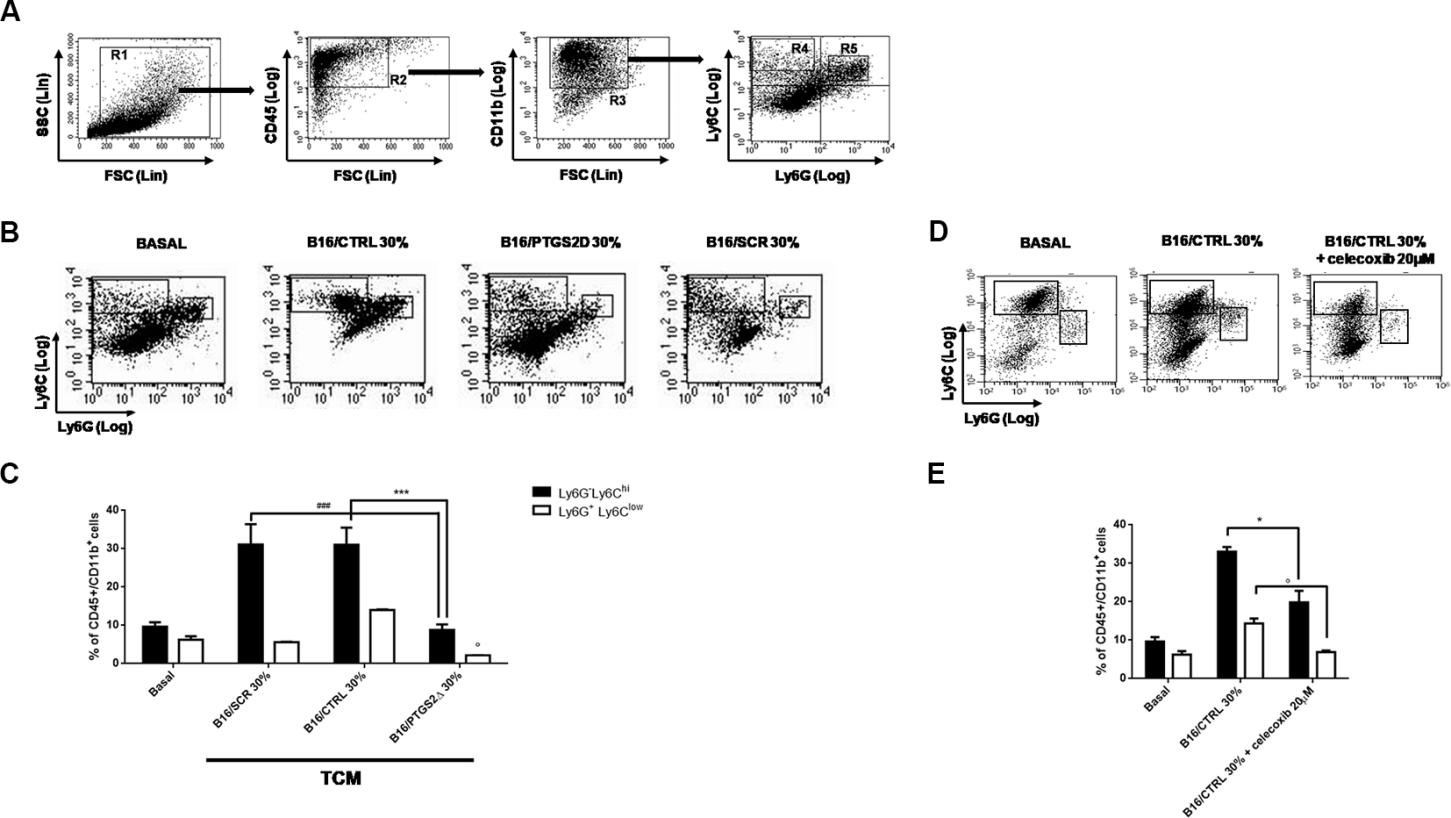
Knockdown of PTGS2 impairs MDSCs differentiation *in vitro*. Wild type bone marrow cells were cultured with GM-CSF and IL-4 alone (BASAL) or with 30% tumor-conditioned medium (TCM) from B16/F10, B16/PTGS2Δ, and B16/SCR cells for 5 days. The surface expression of Ly6G and Ly6C was measured on gated CD45^+^Cd11b^+^ cells by flow cytometry **(A)**. Representative flow cytometry plot **(B)** and relative quantification **(C)** of the two MDSCs subpopulations (R4: Ly6C^hi^Ly6G^−^; R5: Ly6C^low^Ly6G^+^) obtained after stimulation with different TCM. The frequencies of both MDSCs subsets induced by B16/PTGS2Δ-CM was lesser than the frequencies of MDSCs observed with B16/F10-CM and B16/SCR-CM (°*P* < 0.05 vs. B16/CTRL 30%; ***P < 0.001 vs. B16/CTRL 30%; ^###^*P* < 0.001 vs. B16/SCR 30%). Celecoxib (20µM) was added to some wells containing 30% v/v of B16/CTRL TCM at the beginning of the culture **(D**–**E)**. The presence of celecoxib inhibited MDSCs differentiation (*P < 0.05; °*P* < 0.05 vs. B16/CTRL). Data are shown as mean ± SEM of three independent experiments

MDSCs facilitate tumor progression by dampening immunosurveillance and are considered an important target for tumor immunotherapy (Wesolowski et al., 2013). MDSCs are a heterogeneous population of immature myeloid cells identified as CD11b^+^Gr-1^+^. Two different subtypes of MDSCs have been characterized in mice with distinct phenotypes, morphology, and immunosuppressive mechanisms, namely granulocytic MDSCs (gr-MDSCs) and monocytic MDSC (mo-MDSCs) (Youn et al., 2008). Their heterogeneity is tumor dependent and is most likely spawned from the unique inflammatory milieu existing in the TME. To investigate the effect of *ptgs2* knockdown on MDSCs differentiation, we generated MDSCs from murine BM cells by using B16F10 melanoma cells derived CM. Then, we analyzed BM-derived MDSCs using a flow cytometry scheme and markers that have been previously validated for the study of tumor-associated myeloid cells (**Figure 5**) (Youn et al., 2008). We found that culturing BM cells in the presence of B16/CTRL CM led to a strong myeloid differentiation in mo-MDSCs (Ly6C^hi^, Ly6G^−^) and gr-MDSCs (Ly6C^low^, Ly6G^+^). The frequency of mo-MDSCs resulted about threefold higher compared to cells cultured with GM-CSF and IL-4 alone (basal condition) (31.91% vs. 9.74%, *P* < 0.001). Likewise, the frequency of gr-MDSCs was almost doubled than the frequency of gr-MDSCs in basal condition (13.62% vs. 6.59%, *P* < 0.05). These results confirm the importance of tumor-derived factors in inducing MDSCs differentiation. Interestingly, when BM cells were cultured in presence of CM obtained from B16/PTGS2Δ a significant reduction in the frequencies of both mo-MDSCs (9.43% vs. 31.91%, *P* < 0.001) and gr-MDSCs (2.15% vs. 13.62% *P* < 0.05) was observed. Indeed, the frequencies of MDSCs induced by B16/PTGS2Δ-CM was even less than the frequencies of MDSCs observed in basal condition suggesting the key role of PTGS2 in modulating the B16 derived pro-inflammatory factors triggering MDSCs differentiation. B16/SCR-CM had comparable effect of B16/CTRL-CM (**Figures 5B**, **C**). Similarly, addition of the PTGS2 selective antagonist celecoxib (20µM) to B16/CTRL-CM significantly reduced the induction of mo-MDSCs (19.8% vs. 33.04%, *P* < 0.05) and gr-MDSCs (6.91% vs. 19.8% *P* < 0.05) from BM cells, confirming that PTGS2 mediates MDSCs differentiation (**Figures 5D**, **E**).

### *Ptgs2* Knockdown Inhibits Subcutaneous and Metastatic Melanoma in Mice

**Figure 6:**
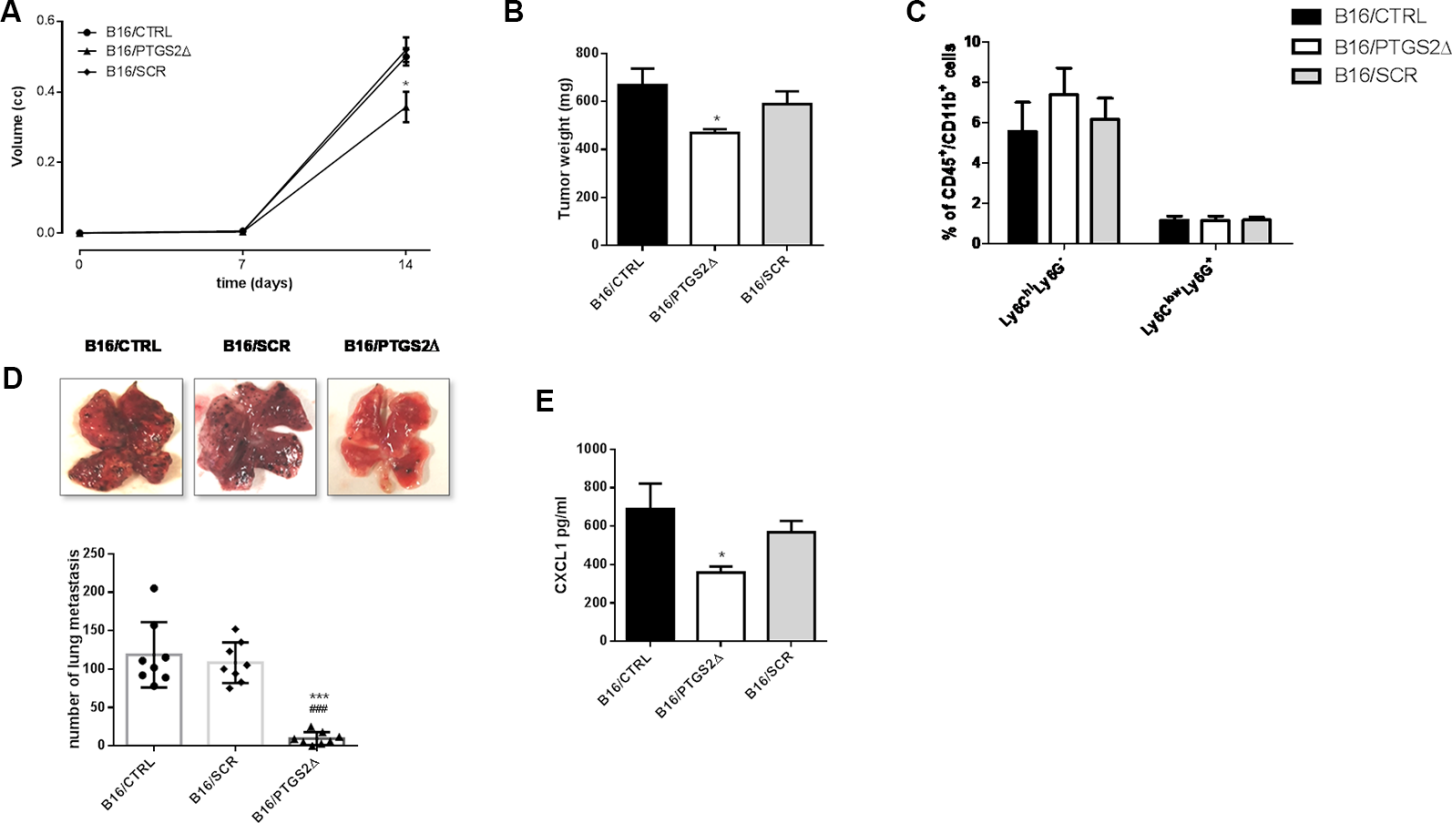
Knockdown of PTGS2 inhibits subcutaneous and metastatic melanoma *in vivo*. B16/F10 (●), B16/PTGS2Δ (▲) or B16/SCR (♦) were subcutaneously implanted in mice. Tumor volume was monitored on the indicated days. The average of tumor volume and tumor weight with SEM is showed respectively in panel **(A** and **B)**. Tumor volume and weight were reduced in B16/PTGS2Δ bearing mice as compared to B16/CTRL and B16/SCR bearing mice (*P < 0.05, n = 8; day 14). Frequency of Ly6G^−^Ly6C^high^ and Ly6G^+^Ly6C^low^ in tumor was assessed by flow cytometry **(C)**. B16/F10 (●), B16/SCR (♦) or B16/PTGS2Δ (▲) were injected *via* the tail vein in mice. Representative macroscopic pictures of mouse lungs, 14 days after inoculation and quantification of the number of metastasis **(D)**. The number of lung metastasis was significantly reduced in B16/PTGS2Δ bearing mice by as compared to B16/CTRL and B16/SCR bearing mice (***P < 0.001 vs. B16/CTRL; *P < 0.001 vs. B16/SCR, n = 8; day 14). CXCL1 plasma levels **(E)** were significantly reduced in B16/PTGS2Δ bearing mice as compared to B16/CTRL bearing mice (^###^*P* < 0.05 vs. B16/CTRL, n = 8; day 14). All data are shown as mean ± SEM.

To evaluate the effect of *ptgs2* knockdown *in vivo*, we subcutaneously implanted B16/PTGS2Δ or B16/CTRL or B16/SCR in C57BL/6 mice. After 14 days, tumor analysis revealed that tumor volume was 30% smaller in B16/PTGS2Δ bearing mice (0.357 ± 0.04 cm3; *P* < 0.05) compared with B16/CTRL and B16/SCR bearing mice (0.500 ± 0.02 cm3; 0.520 ± 0.03 cm3 respectively) (**Figure 6A**). Tumor weight was also reduced in B16/PTGS2Δ bearing mice by 30% (469 ± 15.8 mg mean weight; *P* < 0.05) as compared to B16/CTRL and B16/SCR bearing mice (669 ± 68 mg mean weight; 589 ± 53 mg mean weight respectively) (**Figure 6B**). To verify whether tumor growth inhibition was influenced by the immune cell composition within the TME we determined the frequencies of gr-MDSCs (CD11b^+^Ly6C^low^Ly6G^+^) and mo-MDSCs (CD11b^+^Ly6C^hi^Ly6G^−^) subpopulations in the tumor mass of B16/CTRL, B16/PTGS2Δ, and B16/SCR-bearing mice. However, we did not find a clear difference within the groups (**Figure 6C**).

To deeply investigate on the role of *ptgs2* on melanoma progression, we exploited also a murine model of metastatic melanoma induced following intravenous injection of B16 cells. After 14 days we examined the lungs and we observed an impressive significant reduction of tumor foci in B16/PTGS2Δ-bearing mice compared with B16/CTRL and B16/SCR-bearing mice of about ten-fold (**Figure 6D**).

Epithelial‐to‐mesenchymal transition (EMT) is defined as a program in which cancer cells enhance their migratory and invasive properties in order to trigger metastasis development (Suarez-Carmona et al., 2017). Recently, different studies reported that inflammatory mediators such as cytokines, chemokines, and MMPs can promote cancer cells aggressiveness and the consequent tumor progression (Crusz and Balkwill, 2015; Pesic and Greten, 2016; Revilla et al., 2019,). In particular, CXC chemokine subfamily have been widely associated to cellular transformation, tumor growth, homing, and metastasis. Among these chemokines CXCL1 has been associated with metastatic melanoma (Dhawan and Richmond, 2002). Thus, to translate our *in vitro* findings of *ptgs2* knockdown-reduced migration and invasion into *in vivo* settings, we assessed the CXCL1 plasma levels in tumor bearing mice. As show in **Figure 6E**, serum levels of CXCL1 in B16/PTGS2Δ bearing mice were significantly lower (359 ± 31 pg/ml) than B16/CTRL and B16/SCR bearing mice (689 ± 131 pg/ml; 568 ± 59 pg/ml respectively). This result supports the role of PTGS2 in promoting EMT‐like changes in cancer cells in order to favor tumor progression and metastasis development.

## Discussion

Chemotherapy, targeted therapy and immunotherapy have all been clinically utilized to target melanoma and prolong patient survival. However, most patients do not benefit from such therapies because the genetic features of patient’s tumor influence clinical responses (Garraway, 2013). Thus, the requirement for personalized treatment regimens becomes mandatory (Verma, 2012). Recently, the CRISPR/Cas9 technology has been successfully employed to study gene functions and their role in tumors (Guernet and Grumolato, 2017). In addition, the ability of CRISPR/Cas9 to accurately and efficiently edit genes, not only in cell culture models but also in humans, allows its use as a potentially powerful therapeutic tool in cancer therapy (Martinez-Lage et al., 2018). In 2016, the first clinical trial using CRISPR technology received approval in the United States. This phase I study entitled “Phase I Trial of Autologous T Cells Engineered to Express NY-ESO-1 TCR and Gene Edited to Eliminate Endogenous TCR and PD-1” involves 18 patients with refractory tumors, including melanoma (n = 6), synovial sarcoma (n = 6), and multiple myeloma (n = 6), without effective therapies. The primary endpoint of this trial was to assess the safety and dosage of a novel intervention, as well as the efficacy (https://clinicaltrials.gov/ct2/show/NCT03399448).

Several different gene targets have been identified and proven to be effective against melanoma. Targeting and/or modulating the stromal TME is one strategy to treat melanoma (Roma-Rodrigues et al., 2019). Inflammation underlies this microenvironment and it is considered the strongest supporter in tumorigenesis. Among the different mediators of inflammation, PTGS2 clearly appear to be implicated in cancer progression, by stimulating cell proliferation, cell invasion, inducing vessel formation, and enhancing metastasis and immunosuppression (Sobolewski et al., 2010). Overexpression of PTGS2 has been detected in most types of cancer, including adenocarcinoma, hepatocellular carcinoma, colorectal cancer, breast cancer, pancreatic cancer, and lung cancers (Hashemi Goradel et al., 2019). In the present study, analyzing different gene expression datasets, we demonstrated that PTGS2 is predominately expressed in melanoma tissue while its expression is negligible in normal skin. Previously, we have also demonstrated that PTGS2 level correlates with invasiveness and poor prognosis in melanoma patients (Panza et al., 2016). Both observations indicate that PTGS2 participates in the progression of melanoma. Within the last years, potential therapeutic application of several micro-RNA (miR) that directly regulate PTGS2 expression have been reported in several cancer. We found that miR-143-3p works as tumor suppressor in malignant melanoma. Therefore, decreased levels of miR-143-3p in primary human melanoma lesions and metastases contributes to the up-regulation and overexpression of PTGS2 in melanoma (Panza et al., 2018). Remarkably, full deletion of *ptgs2* in knockout mice markedly reduces melanoma growth (Panza et al., 2016). The evident close association between PTGS2 gene expression and melanoma tumorigenesis (Denkert et al., 2001; Goulet et al., 2003; Lee et al., 2008; Panza et al., 2016; Kim et al., 2019,) pinpoint to PTGS2 as a potential target gene. CRISPR/Cas9-based knockout strategies are increasingly used to analyze gene function. In this study, we used the above technology to further explored the role of PTGS2 in melanoma development and progression. We conducted CRISPR/Cas9 gene-editing strategy to disrupt *ptgs2* gene sequence and eliminate PTGS2 protein expression in B16F10 murine melanoma cells. *Ptgs2* knockdown by CRISPR/Cas9 significantly inhibited cell proliferation as well as migration, invasion, and colony formation of B16F10 melanoma cells. Indeed, when we moved to *in vivo* melanoma models, we found that the absence of *ptgs2* in B16F10 cells dramatically reduces subcutaneous melanoma tumors as well as melanoma metastasis in mice.

PTGS2 is able to induce immune cell recruitment into the tumor tissue to promote an immunosuppressive state in the TME in favor of cancer cell activation (Obermajer et al., 2011). In fact, PTGS2/PGE2 released from cancer cells to this milieu suppresses host immunological responses to tumor‐derived antigens by inducing accumulation of immunosuppressive cells like MDSCs (Sinha et al., 2007). MDSCs are the major component of immune infiltrates in tumors that negatively regulate the anti-tumor immune responses (Gabrilovich and Nagaraj, 2009). Immature MDSCs are generated from BM cells and then they expand and migrate to the TME where a complex milieu of tumor- or stromal-derived factors including vascular endothelial growth factor, IL-6, IL-1β, GM-CSF, transforming growth factor β as well as PGE2 modulate their suppressive function (Bunt et al., 2006; Ezernitchi et al., 2006; Bunt et al., 2007; Sinha et al., 2007). Our findings demonstrate that low- producing PGE2 (B16/PTGS2Δ) cells led to poor differentiation of immature BM cells into mo-MDSCs and gr-MDSCs. However, following subcutaneous engraftment of B16/PTGS2Δ in mice the frequency of intratumoral gr-MDSCs and mo-MDSCs resulted not modified although tumor growth was significantly reduced. These results suggest that PTGS2, at least in part, contributes to create a favorable milieu for MDSCs accumulation. Chemokines are also considered to play a key role since they promote MDSCs migration into the TME (Umansky et al., 2016). The close interplay among these mediators is demonstrated by the up-regulation of CXCL5 and CXCL8 expression in human non-small cell lung cancer cells and of CXCL1 in colorectal cancer cells induced by PTGS2 (Pold et al., 2004; Wang et al., 2006). CXCL1 is also expressed in 70% of human melanomas and it is involved in cellular transformation, tumor growth, homing, and metastasis (Dhawan and Richmond, 2002). Our results, demonstrating that CXCL1 serum levels were significantly reduced in B16/PTGS2Δ melanoma bearing mice, are completely in line with these findings and further support the ability of PTGS2 to regulate CXCL1 secretion in melanoma.

Collectively, these findings confirm that PTGS2 is needed to sustain melanoma progression. Indeed, knockdown of *ptgs2* showed remarkable inhibitory effects on tumorigenic property in melanoma cells, including cell proliferation, colony formation capacity, migration, and invasiveness; and modulated immune response by impairing MDSCs differentiation. Finally, *ptgs2* deletion reduces tumor growth and metastasis formation *in vivo*. Therefore, modulating PTGS2 expression in melanoma cells, perhaps by using genetic engineering like CRISPR technology, could be transformative to improve success rates in the development of new and highly selective drugs for melanoma treatment.

## Data Availability Statement

All datasets generated for this study are included in the article/supplementary material.

## Ethics Statement

The animal study was reviewed and approved by Ministero della Salute Dipartimento per la Sanità Pubblica Veterinaria, la Nutrizione e la Sicurezza degli Alimenti Direzione Generale della Sanità Animale e del Farmaco Veterinario dell’ex Ministero della Salute Ufficio VI - Benessere animale Viale Giorgio Ribotta, 5 EUR Castellaccio 00144 Roma.

## Author Contributions

GE and PC designed, performed the experiments, analyzed the data and wrote the manuscript. VR performed the flow cytometric experiments. GT and GR supervised the flow cytometric analysis. RC and PK performed bioinformatics analysis. AI provided intellectual contributions, supervised all the experiments, critically revised the manuscript and gave final approval to the publication.

## Conflict of Interest

The authors declare that the research was conducted in the absence of any commercial or financial relationships that could be construed as a potential conflict of interest.
